# Berberine suppresses advanced glycation end products‐associated diabetic retinopathy in hyperglycemic mice

**DOI:** 10.1002/ctm2.569

**Published:** 2021-11-04

**Authors:** Ning Wang, Leilei Wang, Cheng Zhang, Hor‐Yue Tan, Yinjian Zhang, Yibin Feng

**Affiliations:** ^1^ School of Chinese Medicine Li Ka Shing Faculty of Medicine University of Hong Kong Hong Kong China; ^2^ Department of Ophthalmology Longhua Hospital Shanghai University of Traditional Chinese Medicine Shanghai China; ^3^ Shanghai Eye Disease Prevention and Treatment Center Shanghai China; ^4^ School of Chinese Medicine Hong Kong Baptists University Hong Kong China

Dear Editor,

The formation of advanced glycation end products (AGEs) and AGEs‐related signalling pathway activation in the retina leads to the initiation of retinopathy in diabetic patients.^1^ In this study, we identified the natural compound berberine (BBR) as a potent AGEs inhibitor that significantly suppressed AGEs formation and its related TLR4/STAT3/VEGF signalling pathway in the retina endothelial cells, thus contributing to its therapeutic effect on diabetic retinopathy (DR).

The hypoglycemic activity of BBR has been intensively reported in both animal models and diabetic patients,^2^ however, in vivo evidence for the protective effect of BBR in DR and the associated mechanism are not lacking. We found that BBR significantly inhibited not only glucose‐induced and also fructose‐induced AGEs formation (Figure 1A,B). To measure the inhibitory effect of BBR on AGEs in vivo, we established streptozotocin‐induced hyperglycemia in mice but not in leptin signalling‐defect db/db or ob/ob mice as we cannot exclude the possible action of BBR on leptin. Streptozotocin‐induced chronic hyperglycemia in mice (Figure S1A), and both AGEs inhibitor aminoguanidine (AGs) and BBR improved fasting blood glucose level and glucose tolerance in hyperglycemic mice (Figure S1B,C), and significantly reduced serum AGEs in a dose‐dependent manner (Figure 1C). BBR was recently found to reduce the AGEs in the lens of diabetic mice.^3^ As AGEs deposition in the retina layer predominately initiates DR, we measured the AGEs level at the retina of hyperglycemic mice. BBR significantly inhibited AGE deposition in the mice's retinas, demonstrating that BBR inhibits the formation and deposition of AGEs in vivo (Figure 1D). In addition, BBR and AGs showed comparable improvement in the blood‐retina (BR) barrier leakage in hyperglycemic mice (Figure 1E). BR barrier breakdown may result from vascular degeneration. By analyzing the retina vasculature (Figure S2A), we found that BBR significantly decreased the endothelial cells/pericytes ratio (Figure S2b) and numbers of acellular vessels (Figure S2C) in hyperglycemic mice. To comprehensively examine the retina condition, we prepared whole‐mount retinas of hyperglycemic mice. An improvement in the typical leakage around the optic nerve head was observed after BBR treatment, as shown by the reduced number of microaneurysms in the retinas (white arrow) in both peripheral fields (Figure 1F). BBR treatment significantly reduced the number of conjunctive vessels per retina (Figure 1G), the area covered by vessels (Figure 1H), and the number of microaneurysms in the flat‐mounted retinas (Figure 1I).

**FIGURE 1:**
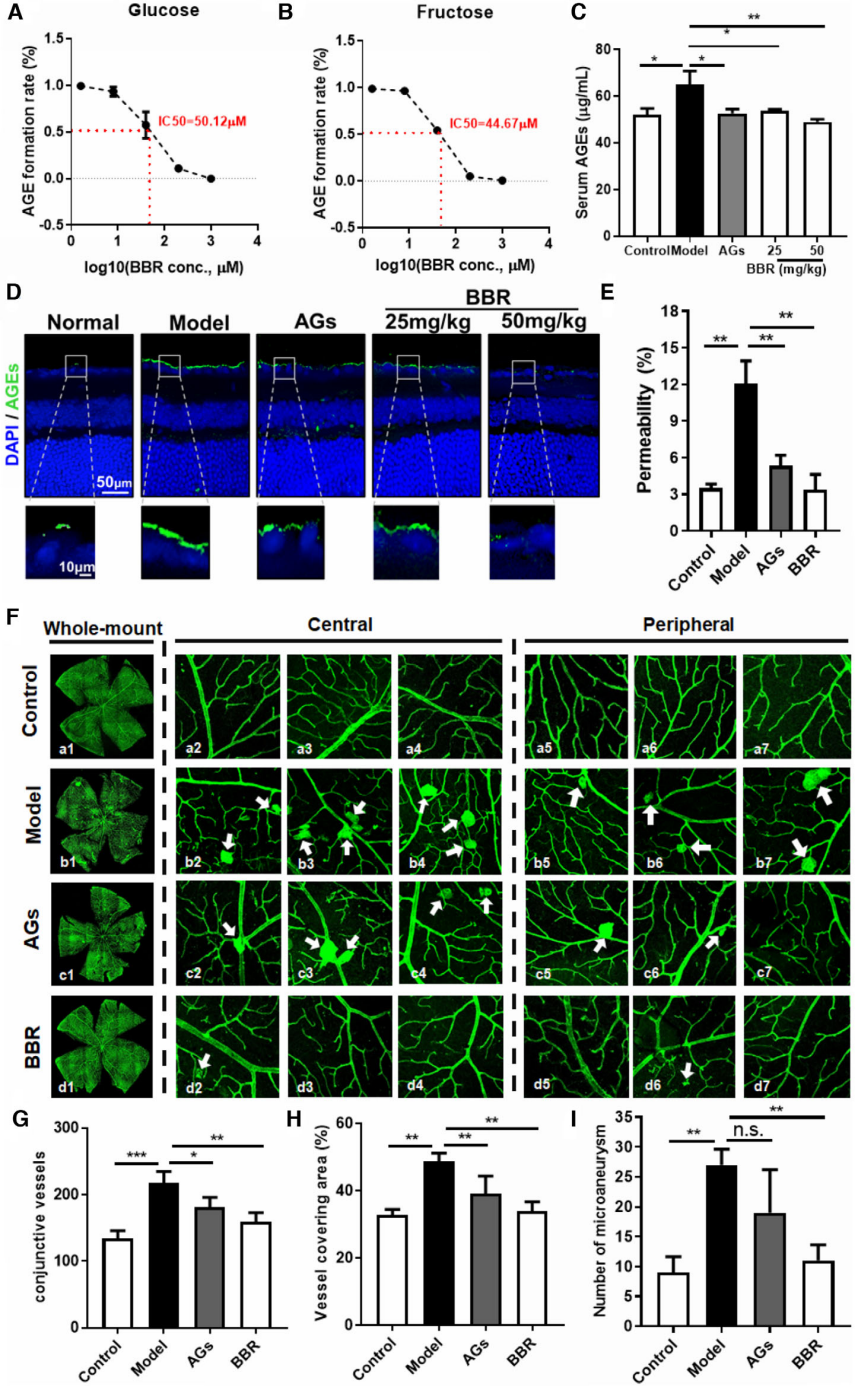
Berberine suppressed advanced glycation end products (AGEs) formation and diabetic retinopathy (DR) in hyperglycemic mice. (A) Sterile 50 mg/ml bovine serum albumin and 0.2 M phosphate‐buffered saline were incubated with 144 mg/ml glucose in the presence of 1.6, 8, 40, or 200 μM berberine (BBR) for 7 days at 37°C. Fluorescence was measured on a luminescence spectrometer at an excitation wavelength of 370 nm and an emission wavelength of 440 nm. BBR dose‐dependently suppressed AGE formation by glucose. (B) Sterile 250 mM fructose and 0.2 M PBS were incubated for 6 days at 37°C. Fluorescence was measured on a luminescence spectrometer at an excitation wavelength of 370 nm and an emission wavelength of 440 nm. BBR dose‐dependently suppressed AGE formation by fructose. (C) Serum was collected from streptozotocin (STZ)‐induced hyperglycemic mice (*n* = 5) and the AGE content in the serum was detected by ELISA. Treatment with either AG or BBR significantly suppressed serum AGE levels in hyperglycemic mice. (D) Whole eyeballs were collected from hyperglycemic mice and fixed. Paraffin sections (5 μm) were prepared, stained with an anti‐AGE antibody, and imaged under a confocal microscope. Treatment with either AG or BBR significantly suppressed retinal AGE levels in hyperglycemic mice. (E) Hyperglycemic mice were intravenously injected with 45 mg/kg Evans blue 3 h prior to sacrifice. The retinas were isolated and Evans blue deposited on the retina was extracted, dissolved in dimethylsulphoxide, and the absorbance was measured at 620 nm. The percentage of circulating Evans blue that leaked onto the retina was calculated. Treatment with either AG or BBR significantly reduced the retinal leakage of Evans blue in hyperglycemic mice. (F) Retinal whole mounts were prepared and labelled with isolectin‐B4 (Green). The images were captured using a confocal microscope. Central and peripheral fields of the whole‐mount retinas were captured. Microaneurysms in retinas are indicated with arrows. Treatment with either AG or BBR showed a significant improvement in the retinal vasculature in hyperglycemic mice. The number of conjunctive vessels, the area covered by vessels, and the number of microaneurysms were determined using AngioTool. AG or BBR treatment significantly suppressed the number of conjunctive vessels (G), reduced the area covered by vessels, (H) and the number of microaneurysms (I) in the retinas of hyperglycemic mice. *p < 0.05, **p < 0.01, ***p < 0.001 when compared to the model group

Increased retinal neo‐vasculature may result from endothelial cell hyperactivation by excessive AGEs.^4^ At non‐toxic concentrations (Figure 2A), BBR exhibited significant dose‐dependent repression of AGE‐induced proliferation (Figure 2B), leakage of FITC‐dextran from the monolayer (Figure 2C), wound closure (Figure 2D), and transmembrane migration (Figure 2E) of human retina endothelial cell line (HRECs). VEGF stimulates vascular endothelial cell proliferation, migration, and vasopermeability in the retina.^5^ A significant increase in VEGF production was found in AGEs‐treated HRECs, and this was effectively suppressed by both AGs and BBR treatment (Figure 2F). This was consistently observed in retinal extracts of hyperglycemic mice, and high‐dose BBR treatment showed stronger inhibition on retina VEGF production in hyperglycemic mice (Figure 2G). A previous study showed that AGEs deposited in the retina bind to RAGE in endothelial cells, activating TLR4 expression and pathways responsible for VEGF production.^6^ BBR treatment significantly inhibited the AGE‐induced activation of TLR4 and downstream STAT3 signalling in HRECs cells (Figure 2H) and in the retinas of hyperglycemic mice (Figure 2I). To determine whether the inhibitory effect of BBR on VEGF production was mediated by its inhibition of STAT3, we transfected a plasmid encoding constitutively activated STAT3 mutant (STAT3C) into BBR‐treated HRECs. Transfection of STAT3C significantly recovered STAT3 activity in AGEs‐treated HRECs in the presence of BBR (Figure 2J). Moreover, constitutive activation of STAT3 in these cells significantly restored the VEGF production (Figure 2K). These observations suggest that inhibition of VEGF production by BBR was indirectly dependent on activation of STAT3 in AGEs‐treated endothelial cells.

**FIGURE 2:**
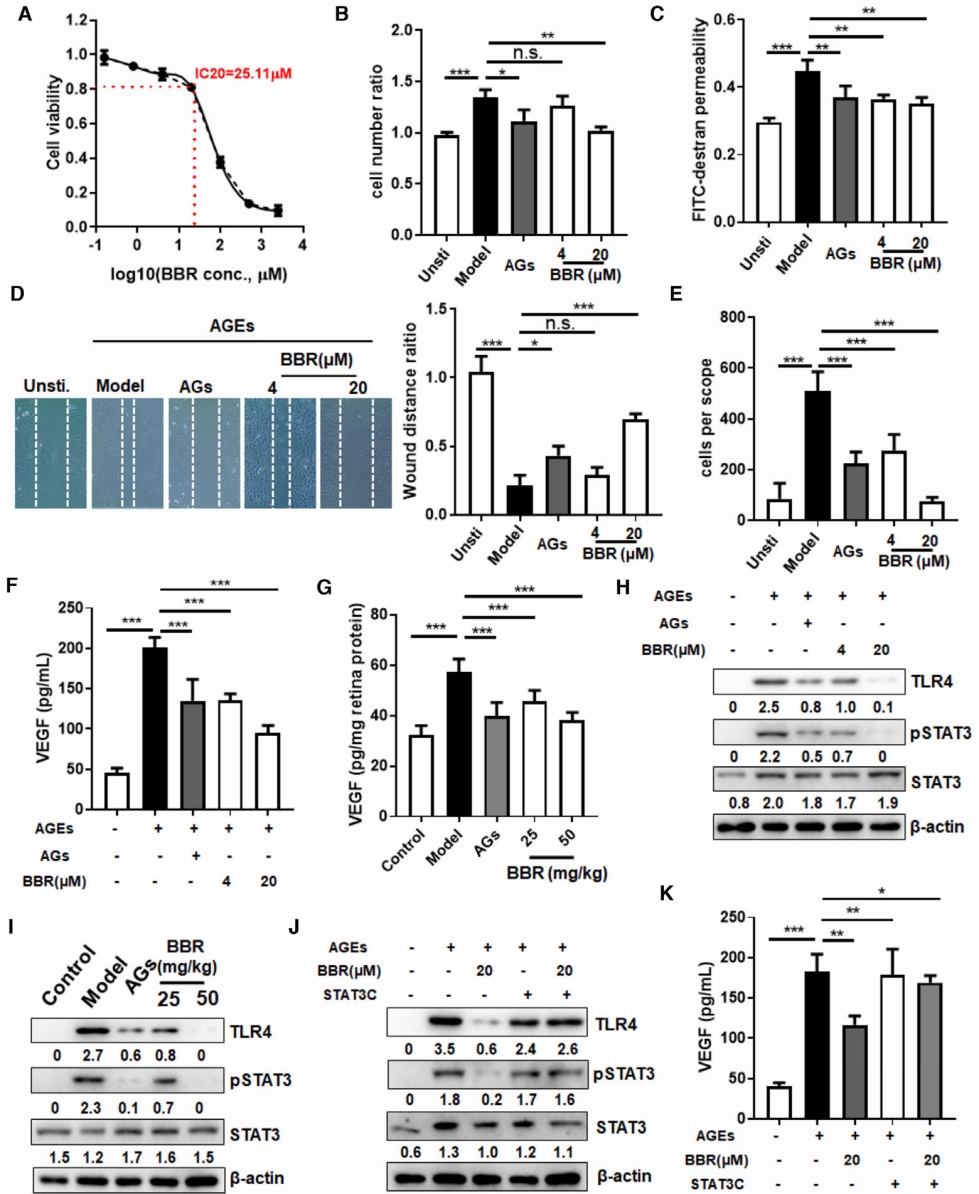
Berberine suppressed retina endothelial cell activation through advanced glycation end products (AGEs)/TLR4/STAT3/VEGF signalling. (A) Human retina endothelial cell lines (HRECs) were cultured with different doses of berberine (BBR) in the presence of 20 μg/ml AGEs for 24 h. The cytotoxicity of BBR on HREC cells was measured by MTT assay and the inhibitory concentration (IC)_20_ of BBR was calculated as 25.11 μM. Doses of BBR lower than the IC_20_ were considered non‐toxic to HREC cells. (B) HRECs (1 × 10^4^) were cultured in the presence of 20 μg/ml AGEs in combination with various treatments. Cells with no AGE treatment were considered as unstimulated cells (Unsti.). Seventy‐two hours after incubation, the number of cells was counted and the fold increase was calculated. AG (20 μg/ml) and BBR (20 μM) significantly inhibition HREC proliferation induced by AGEs. (C) HRECs (1 × 10^5^) were seeded onto transwell inserts with a pore size of 0.4 μm (Costar; Corning Inc., Corning, NY, USA) until reaching full confluence. Two hundred microlitres of extracellular matrix (ECM) with 1% FBS, 25 μg/ml FITC‐dextran and 20 μg/ml standard AGEs, alone or in combination with BBR (4 or 20 μM), were applied to the top inserts, whereas 900 μl of ECM with 1% FBS was added to the lower chambers and the plates were incubated for 24 h. Culture media at the upper and lower chambers were then collected for fluorescence measurement with an excitation at 494 nm and emission at 518 nm. The amount of FITC‐dextran leakage was then calculated. AG and BBR significantly inhibited the permeability of the AGE‐treated HREC monolayer to FITC‐dextran. (D) HRECs were seeded at the full confluence and a line was scrapped in the middle of the culture wells. Cells were then incubated with 20 μg/ml AGEs, in combination with various treatments, for 24 h. The image was captured and the wound closure rate was calculated. BBR and AG showed potent inhibition of AGE‐induced wound closure of HRECs. (E) HRECs (1 × 10^5^) were seeded onto the transwell inserts (pore size, 8 μm) supplemented with 20 μg/ml AGEs in combination with various treatments. Cells passing through the transwell membrane within 4 h were stained with crystal violet and quantified under a light microscope. BBR and AG potently inhibited AGE‐induced migration of HRECs through the transwell membrane. (F) Conditioned medium was collected from HREC cultures in the presence of 20 μg/mL AGEs in combination with various treatments. VEGF concentration was determined by ELISA. AG and BBR treatment potently suppressed VEGF production by AGE‐treated HRECs. (G) Serum was collected from hyperglycemic mice after different treatments. VEGF concentration was determined by ELISA. AG and BBR treatment potently suppressed serum VEGF levels in hyperglycemic mice. Total protein was collected from AGE‐treated HRECs (H) or homogenized retinas (I) after various treatments. Immunoblotting was performed to determine the levels of TLR4, phosphor‐STAT3, and STAT3. β‐actin was used as an internal control. Treatment with AG or BBR significantly suppressed TLR4 expression and phosphor‐STAT3 activity in both HRECs and retinas from hyperglycemic mice. HRECs were transfected with a plasmid expressing a constitutively phosphorylated STAT3 mutant 48 h prior to stimulation with 20 μg/mL AGEs. Cells then underwent various treatments for 24 h, after which total protein (j) and conditioned media (K) were collected. The levels of TLR4, phosphor‐STAT3, and STAT3 were determined by immunoblotting and VEGF production was quantified by ELISA. The recovery of TLR4 expression and VEGF production suppressed by BBR in AGE‐treated HRECs was observed. *p < 0.05, **p < 0.01, ***p < 0.001 when compared to the model group in mouse experiments, and to AGE‐treated groups in cell experiments

To further understand the mediating role of TLR4/STAT3 suppression in the inhibitory effect of BBR on retina VEGF expression and endothelial activation, AGE‐induced endothelial cells were co‐treated with BBR and the TLR4 agonist, lipopolysaccharide (LPS). The presence of LPS significantly restored TLR4/STAT3 (Figure 3A) and recovered the mRNA and protein expression levels of VEGF in BBR‐treated activated HRECs (Figure 3B,C). Consistently, inhibition of cell proliferation, vasopermeability, wound closure, migration, and transmembrane invasion of AGEs‐treated HRECs by BBR was partially recovered by the presence of LPS (Figure S3A–D). We then supplemented BBR‐treated hyperglycemic mice with an intraperitoneal injection of LPS. LPS significantly abolished the hypoglycemic effect of BBR (Figure S4A,B). Retinal vascular samples were prepared to quantify the endothelial cells, pericytes, and acellular vessels (Figure S4C). Supplementation with LPS completely abolished the inhibitory effect of BBR on E/P ratio (Figure S4D) and acellular vessel formation (Figure S4E). This was further evidenced by Evans blue leakage experiment, in which LPS supplementation was shown to preserve the BBR‐induced improvement in retinal leakage in hyperglycemic mice (Figure 3D). Furthermore, analysis of whole‐mount retinas showed that LPS treatment significantly increased the vascular leakage in BBR‐treated hyperglycemic mice (Figure 3E). The effect of BBR on the conjunctive vessels per retina (Figure 3F), vessel covered area (Figure 3G), as well as number of microaneurysms (Figure 3H) in the flat‐mounted retinas were attenuated in the presence of LPS.

**FIGURE 3:**
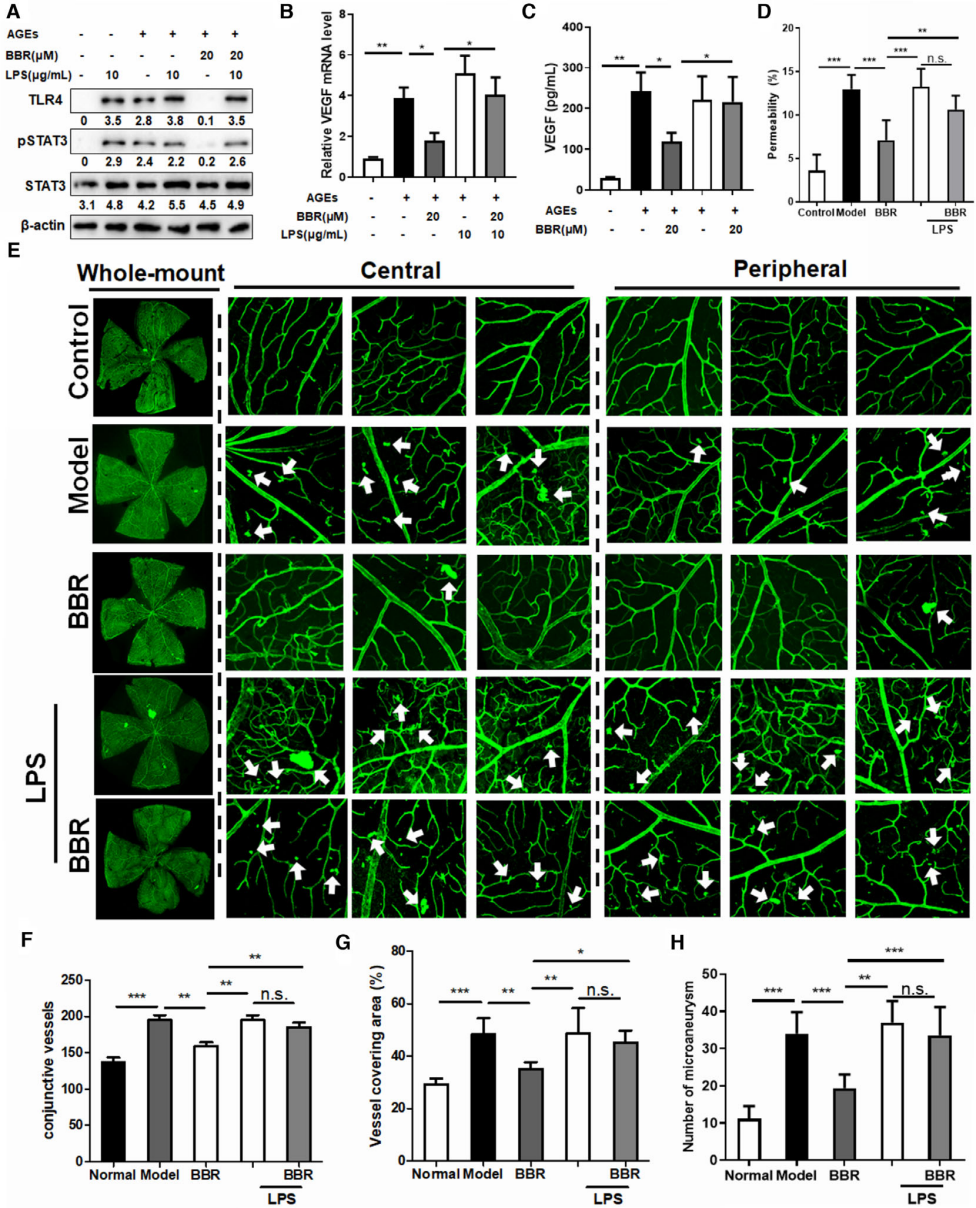
Inhibition of advanced glycation end products (AGEs)‐induced TLR4/STAT3/VEGF signalling is responsible for the improvement of diabetic retinopathy (DR) by berberine in hyperglycemic mice (A) Human retina endothelial cell line (HRECs) were pre‐treated with 100 μg/ml lipopolysaccharide (LPS) for 30 min. The medium was then washed away, and cells were treated with berberine (BBR) in the presence of 20 μg/ml AGEs for 24 h. The levels of TLR4, phosphor‐STAT3, and total STAT3 in HREC cells (a), the mRNA expression levels of VEGF (B), and VEGF levels in the conditioned media (C) were measured by immunoblotting, quantitative real‐time PCR, and ELISA, respectively. LPS pre‐treatment recovered TLR4 expression in BBR‐treated HRECs and restored the mRNA and protein expression levels of VEGF. (D) Hyperglycemic mice (*n* = 5) were intraperitoneally injected with a single dose of LPS (3 mg/kg) immediately before BBR treatment for 8 weeks. The percentage of circulating Evans blue that leaked onto the retina was calculated. Pre‐treatment with LPS significantly restored Evans blue leakage onto the retina in BBR‐treated hyperglycemic mice. (E) Retina whole‐mounts were prepared and labelled with isolectin‐B4 (Green). The overall image of the retina whole‐mount was captured by a confocal microscope. Central and peripheral fields of the whole‐mount were captured. Microaneurysms in the retinas are indicated with arrows. Pre‐treatment with LPS significantly abolished the improvement in the condition of the retina in BBR‐treated hyperglycemic mice. The number of conjunctive vessels, the area covered by vessels, and the number of microaneurysms were determined using AngioTool. Pre‐treatment with LPS significantly abolished the improvements in the number of conjunctive vessels (G), the area covered by vessels (H), and the number of microaneurysms (I) in the retinas of hyperglycemic mice treated with BBR. *p < 0.05, **p < 0.01, ***p < 0.001 compared to the model group in mouse experiments, and to AGE‐treated groups in cell experiments

AGEs signalling might serve as a novel therapeutic target in DR. Aminoguanidine was proven to significantly improve the experimental DR,^7^ but has not been approved for clinical treatment because of its potential adverse effects.^8^ In our study, BBR was found to be a novel inhibitor of AGE formation in vitro and in vivo. At a low dose, BBR treatment suppressed DR by inhibiting AGE/RAGE signalling in the retina, whereas at higher doses, BBR may directly suppress blood glucose levels, which may, subsequently, suppress AGE formation, improve the overall diabetic condition, and thus, relieve DR. Considering the safety of BBR, as determined by extensive clinical studies,^9^ our results shed light on its use as a hypoglycemic alkaloid and an effective AGE inhibitor for the management of DR (Figure S5).

## CONFLICT OF INTEREST

The authors declare that they have no conflict of interest.

## Supporting information



Supporting Information
