# Supplementary material for: Berberine suppresses advanced glycation end products‐associated diabetic retinopathy in hyperglycemic mice
Source: Clin Transl Med. 2021 Nov 4;11(11):e569. doi: 10.1002/ctm2.569 (PMC8567055; doi:10.1002/ctm2.569)
Supplement: Supplementary file 1 — Supporting Information [file CTM2-11-e569-s001.docx]

**Supplemental Materials**

**Materials and Methods**

***Drugs and chemical reagents***

Berberine (BBR) is a natural alkaloid derived from a variety of medicinal plants including Coptis Rhizome. It has been reported with various pharmacological effects such as anti-inflammation, anti-oxidation, hepatic protection and anti-cancer^1-3^. The hypoglycemic activity of BBR was intensively reported by a series of studies , the findings of which conclude a remarkable blood glucose-reducing effect of BBR in both animal models and patients of type 2 diabetes ^4 5^. Meanwhile, fasting blood glucose of non-obese diabetic (NOD) mice ^6^, which spontaneously develop Type 1 diabetes, was decreased by BBR. The protective effect of BBR on diabetic nephropathy was recently gained a lot of attentions ^7 8^. Recent studies showed BBR can protect retinal endothelial cells from attack by leukocytes, which receded capillary degeneration in diabetic patients ^9^. Berberine chloride hydrate (Cat. No.14050), Streptozotocin (Cat. No.S0130), Aminoguanidine (Cat. No. 396494), Evans Blue (Cat. No.E2129), Formamide (Cat. No.F9037), FITC-Dextran (40k) (Cat. No.FD40), Thiazolyl Blue Tetrazolium Bromide (MTT, Cat. No.M2128), Lipopolysacchride (LPS, Cat.No. L2630) and Crystal Violet solution (Cat. No.V5265) were purchased from Sigma-Aldrich (Hong Kong). Trypsin (1:250) (Cat. No.0458) used for retinal digestion was obtained from Amresco (USA). Advanced glycation end products-BSA (Cat. No.121800) was obtained from Millipore. Serum AGEs Elisa kit was purchased from USCN (Cat. No.CEB353Ge, China). Anti-AGEs (Cat. No.ab23722) and anti-TLR4 (Cat. No. ab22048) antibodies were obtained from Abcam (UK). Anti-isolectin-IB4 (Cat. No. I21411) was purchased from Thermo scientific (USA). Anti-phosphor-STAT3 (Cat. No. 9145), anti-STAT3 (Cat. No. 12640) and anti-β-actin (Cat. No. 3700) were purchased from Cell Signaling Technologies (USA). Human retinal endothelial cell line (Cat. No.6530) and endothelial cell media (Cat. No.1001) were purchased from Sciencell. All the reagents were strictly kept as instructed. Streptozotocin and MTT were specially kept out of light.

***Animals***

6- to 8-week male C57BL/6J mice was housed at Center for Comparative Medicine Research (CCMR) of the University of Hong Kong (HKU). The mice were given sufficient pellet diet, water and kept at 12-h light/dark cycle. Protocol of the animal experiments were approved by the Committee on the Use of Live animals in Teaching and Research (CULATR) of HKU (Certificate No.: 3354-14).

***Streptozotocin-induced DR model and interventions***

Mice weighting 22 to 25g were administrated with five consecutive doses of Streptozotocin (STZ) (55mg/kg in citrate buffer, pH=4.5) via intraperitoneal injections to induce diabetic retinopathy. Normal groups of mice were given same volume of citrate buffer. Four days after the last STZ injection, blood glucose was measured with glucometer (Roche, accu-chek active). STZ-treated mice with blood glucose ranging from 16.0 to 25.0 mmol/dl were deemed as hyperglycemic mice and were randomly divided into groups. It was followed by 8-week oral gavage interventions of different treatments. Mice in normal control and model groups (n=8) were treated with ddH_2_O, the positive control group of mice (n=8) were fed with aminoguanidine (AGs) (100 mg/kg) every day, while BBR-treated mice (n=8) were daily treated with 25mg/kg (low dose) and 50 mg/kg (high dose) of BBR respectively. For supplementing LPS in mice, hyperglycemic mice with or without BBR treatment was given a single i.p. dose of LPS injection (3 mg/kg) at the beginning of experiment accordingly to our previous study ^10^.

***Measurement of random, fasting blood glucose and glucose tolerance***

Random blood glucose (RBG) were measured every week, while fasting blood glucose (FBG) and glucose tolerance test (GTT) was performed at the end of the study. To measure GTT, intraperitoneal injection of sterile glucose (2g/kg) was performed following 5-h fasting, and then blood glucose was measure at 0, 30, 60, 90 and 120 min after glucose injection. The area under the curve was calculated as an index of glucose tolerance.

***Retinal vascular degeneration assessment***

Fresh eyeballs were fixed in 4% PFA for 6 h, and then retina was isolated and slightly rinsed with PBS buffer. 3% of trypsin dissolved in 0.1M Tris buffer (pH=8.2) was used to digest the retinas with gentle shaking for one to two h in 37℃. When retinal debris appeared, the incubation was terminated and non-vascular sections were washed away with progressed syringe filled with PBS. The reserved retina vasculature was then subject to hematoxylin-eosin staining and imaged under a microscope (LEICA) with a digital camera (CoolSNAP). Areas of microscope were randomly selected from 8 random fields (0.076mm^2^ for each) from four angles of the retina (0°, 90°, 180°, and 270°), in which 4 sections at both 1mm and 2mm from the optic nerve head have been selected respectively. The quantification of endothelial cells/ pericytes and acellular capillaries have been measured by AngioTool (NIH, USA). Each sample was investigated by two individual researchers.

***Retinal permeability assessment***

Retinal permeability was quantified with Evans blue leakage. Briefly, mice were anaesthetized with Ketamine/Xylazine (100mg/kg and 10mg/kg). Then Evans blue with the dose of 45mg/kg in 0.9% saline buffer was injected intravenously to allow circulation for 3 h so that sufficient permeation through broken BR barrier incurred. 0.5ml blood from heart of mice was collected and mice were then perfumed with citrate buffer (0.05M, pH 3.5) to clear the remaining dye. Retinas then were isolated and dried at 60℃ for 5 h. The dried retina was weighed before being steeped in 120μl formamide at 70℃ for 18 h. Then the mixture was centrifuged down for 90min at 14000 rpm at 4℃. Supernatant was collected and absorbance at 620nm was measured. Standard curve was created so that concentration of the dye in samples could be absolutely calculated. Permeability dysfunction was calculated as following and showed as µl plasma $\times$g retinal dry wt^-1^· h^-1^.`

$$\frac{Evans blue (\mu l)/(Retina dry weight g)}{Time averaged Evans blue concentration:(\mu g)/(Plasma \mu l\times Circulation time h)}$$

***Retina wholemounting***

Retinal vasculature and microaneurysms were visualized by immunofluorescence. Briefly, mouse was anesthetized via intraperitoneal administration of 200 mg/kg pentobarbital. Retina was collected and then fixed with 4% PFA for 12 h followed by post-permeabilization in 70% ethanol for 2 h. Four cuts were made to flatly lie the whole retina. Then, retina was rinsed in PBS followed by incubating in 3% Triton X100 for 2 h. Afterwards, retinae were moved to the blocking solution and incubate 2 h, which consists of 10% goat serum and 0.3% Triton X100. After that, retina was stained with 0.02mg/ml isolectin GS-IB4 (Invitrogen, USA) for 48 h. When the immunostaining was finished, the retina was placed via retinal ganglion cell layer up for capturing the representative figures. More than three fields were randomly selected from four angles (0°, 90°, 180°, and 270°) of the whole retina, in which 1mm or 2mm from the optic nerve have been defined as peripheral and central region of the retina, respectively. AngioTool was adopted to quantitatively measure the condition of vasculature in whole mount retinal tissues. Meanwhile, ImageJ was applied for counting the retinal microaneurysm. All the representative figures were taken by a confocal laser microscope with 200× magnification (Carl Zeiss LSM 780, USA).

***Formation of advanced glycation end products with D-fructose and D-glucose***

Inhibitory effect of BBR on AGEs formation were assessed in the system composed of either D-fructose or D-glucose. Reagents were sterilized before be used. Glucose-derived AGEs were produced by incubating 144mg/ml glucose, 50mg/ml BSA (Sigma, USA), and 0.2M PBS for 7 days at 37°C, while fructose-derived AGEs were produced by incubation in combination of 10mg/mL BSA, 250mM fructose and 0.2M PBS for 6 days at 37°C. 1.6, 8, 40, 200µM BBR were added into different groups right before incubation. Study was performed in triplicate. Fluorescence intensity was measured at 370nm (excitation) and 440nm (emission) on a LS55 Luminescence Spectrometer (PerkinElmer, Germany). Data with rejection of background was recorded.

***Cell culture***

Human retinal endothelial cell (HRECs, ScienCell, USA) line was cultured in Endothelial Cell Medium (ECM, Sciencell) supplemented with 5% fetal bovine serum (FBS), 1% penicillin/streptomycin, and 1% endothelial cell growth supplement (ECGS) in a humid environment at 37℃, 5% CO_2_. For LPS-related study, cells were pretreated with 100µg/ml for 30min. Medium was then washed away for additional treatments.

***Cytotoxicity test***

MTT assay was used to assess toxicity of BBR on HRECs. Briefly, HRECs at the density of 1.0$\times$10^4^/well was seeded into 96-well plate then BBR in the dose of 0.16, 0.8, 4, 20, 100μM were added in the presence of 20µg/ml AGEs for incubation for 24 h at 37°C, 5% CO_2_. By the last 4 h of incubation, 10$\mu L$ MTT (5mg/mL) was added to each well. The formed crystals were dissolved with 100$\mu L$ DMSO and the absorbance was measured at 575nm by a microplate reader (Labsystems, Finland).

***Measurement of HREC permeability***

1x10^5^ HRECs were seeded onto Transwell insert with pore size of 0.4µm (Corning Costar, USA) with full confluence. 200µl ECM with 1% FBS, 25µg/ml FITC-Dextran and 20µg/ml standard AGEs alone or in combination with BBR (4µM or 20µM) were applied to the top inserts respectively, whereas 900 µl ECM with 1% FBS were added in the lower chambers. After 24 h of incubation, 100μl of medium was collected from both upper and lower chambers and the fluorescence intensity was measured with 494nm (excitation) and 518nm (emission) on a Luminescence Spectrometer. HRECs monolayer permeability was calculated as follows, *permeability*(%*) =solution signal from lower chamber*$\times$ 9*/ (solution signalling from lower chamber*$\times$ 9 *+ solution signal from upper chamber*$\times$2*)*$\times$ 100%. Assays were performed in triplicate.

***Wound Closure assay***

HRECs were cultured on 12-well plate until confluence. After starved for 2 h In FBS-free and ECGs-free ECM, a line was scraped at the middle of culture well using a pipette tips and cells further incubated with 20µg/ml AGEs alone or in combination with BBR (4µM or 20µM) for 24 h. Image was capture under a digital microscope (AMG, EVOS) with 40X magnification. The closure of the wound which represented the ability of cell migration was quantified.

***Migration assay***

1$\times$10^5^ HRECs were seeded onto the transfer inserts (pore size as 8µm, BD Falcon, USA). These cells were supplemented with 400µl of free ECM with or without 20µg/ml AGEs in presence or absence of BBR (4µM or 20µM). After 4 h of incubation, cells on the upper chambers were removed with cotton swabs and the inserts were fixed with 4% PFA for 30min. Migrated cells were stained with Crystal Violet solution and image was captured with a digital microscope (AMG, EVOS) at 40x magnification and quantified.

***Concentration of serum AGEs***

Serum AGEs were assessed with ELISA kit according to the manufacturer's instruction. Each sample was conducted in triplicate. Briefly, 50$\mu l$ serum was incubated with 50$\mu l$ detection reagent A for 1h, followed by incubation with reagent B for 30min and then 90$\mu l$ substrate solution for 20 min consecutively. All the incubations were performed at 37℃, and 3-time wash were needed at each interval. After incubation, 50$\mu l$ stop solution was added. Absorbance at 450nm was read by a microplate reader.

***Immunohistochemistry***

The 7µm-thick sections of eyecups were used to assess the expression and localization of retinal AGEs. Briefly, PBS solution containing 5% serum and 0.3% Triton was used to block and permeate samples for 2 h. The slides were incubated with appropriate antibodies (1:100) at 4°C for 48 h, followed by secondary antibody staining in dark for 2 h. DAPI was used as counter stain. Image was captured under a fluorescence microscope (Carl Ziess, USA, 20X, CCD camera). ***Immunoblotting***

Retina tissues and HRECs were lysed and protein was collected by centrifugation. Protein was quantified and equal amount of protein was loaded on 7.5% to 12.5% SDS-PAGE for electrophoresis before transferring onto a polyvinylidene fluoride (PVDF) membrane. The membrane was then blocked with TBST buffer containing 5% BSA at room temperature for 2 h, followed by primary antibodies incubation overnight at 4°C. The membrane was then incubated with appropriate secondary antibody at room temperature for 2 h and washed. Immunoreactivities were measured with ECL select as substrate (GE Healthcare, Germany) and captured by a chemiluminescence imaging system (Bio-Rad, USA).

***2.15 Statistics***

In vitro experiments were conducted in triplicate. Data were analyzed by Prism 8.0 and expressed as mean$\pm$standard derivation (SD). Data according with normal distribution were assessed by an ordinary ONE-WAY ANOVA, whereas those were not met with normal distribution were evaluated by Kruskal-Wallis test. Outcomes with p<0.05 illustrated statistical significance.

**Supplemental Figures**


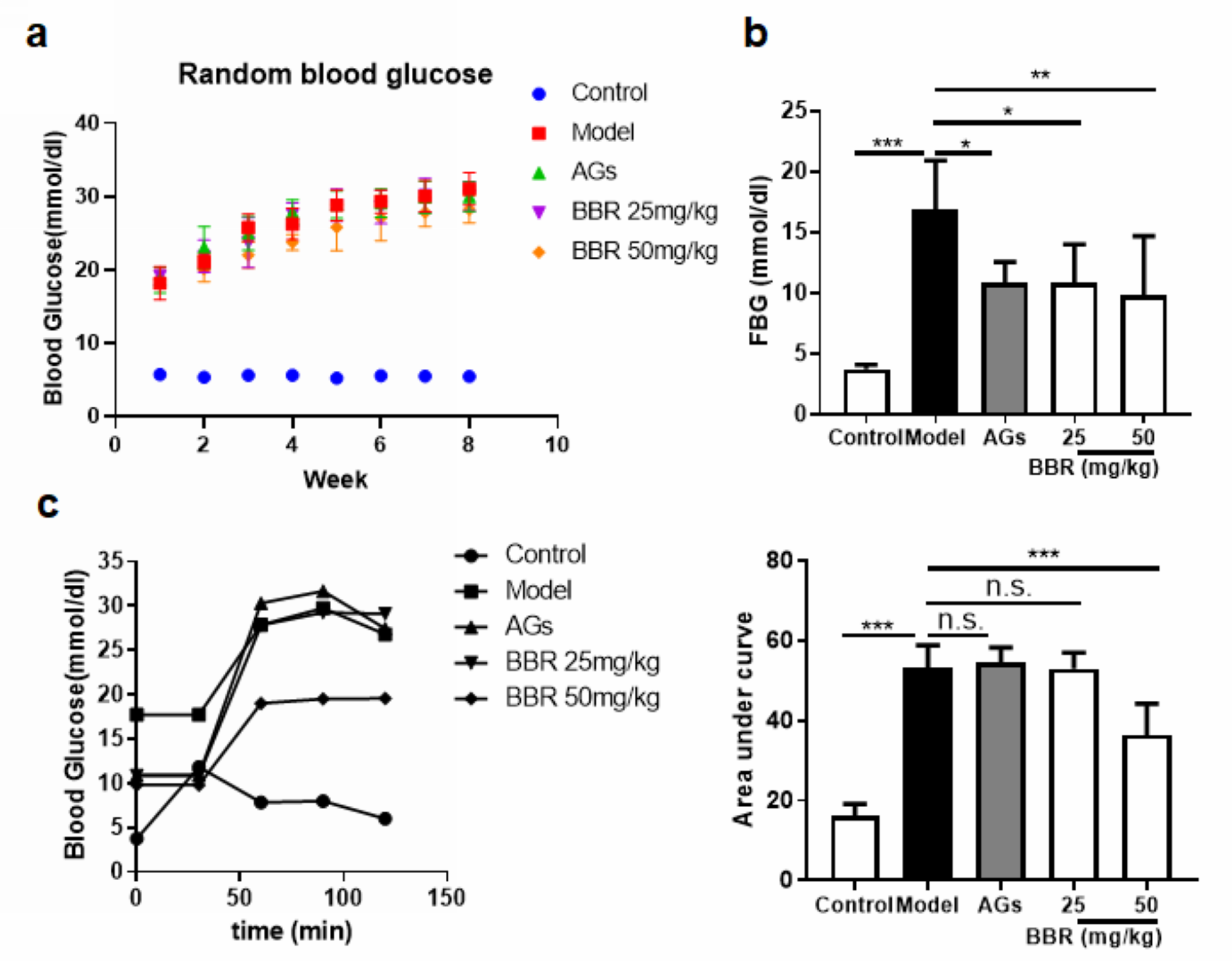


**Figure S1 High dose of berberine suppressed hyperglycemia in hyperglycemic mice**

**a.** Random blood glucose of STZ-induced hyperglycemic mice (n=5) with various treatment was taken weekly. It was shown that STZ injection caused a stable hyperglycemia in C57/BL/6J mice within 8 weeks, and only high dose treatment of BBR suppressed blood glucose in these mice; **b.** Fasting blood glucose (FBG) was measured at the end of 8-week treatment in hyperglycemic mice with 5 h starvation. It was shown that both AGs and BBR treatment can improve FBG in hyperglycemic mice. **c.** At the end of 8-week treatment, hyperglycemic mice were starved for 5 h and 2 g/kg glucose was i.p. injected to the mice. the blood glucose was tested at 0, 30, 60, 90 and 120 min post glucose injection. Curve was drawn and the area under curve (AUC) was quantified. It was shown that only high dose of BBR treatment can improve the glucose tolerance in hyperglycemic mice. *p<0.05, **p<0.01, ***p<0.001 when compared to model group.


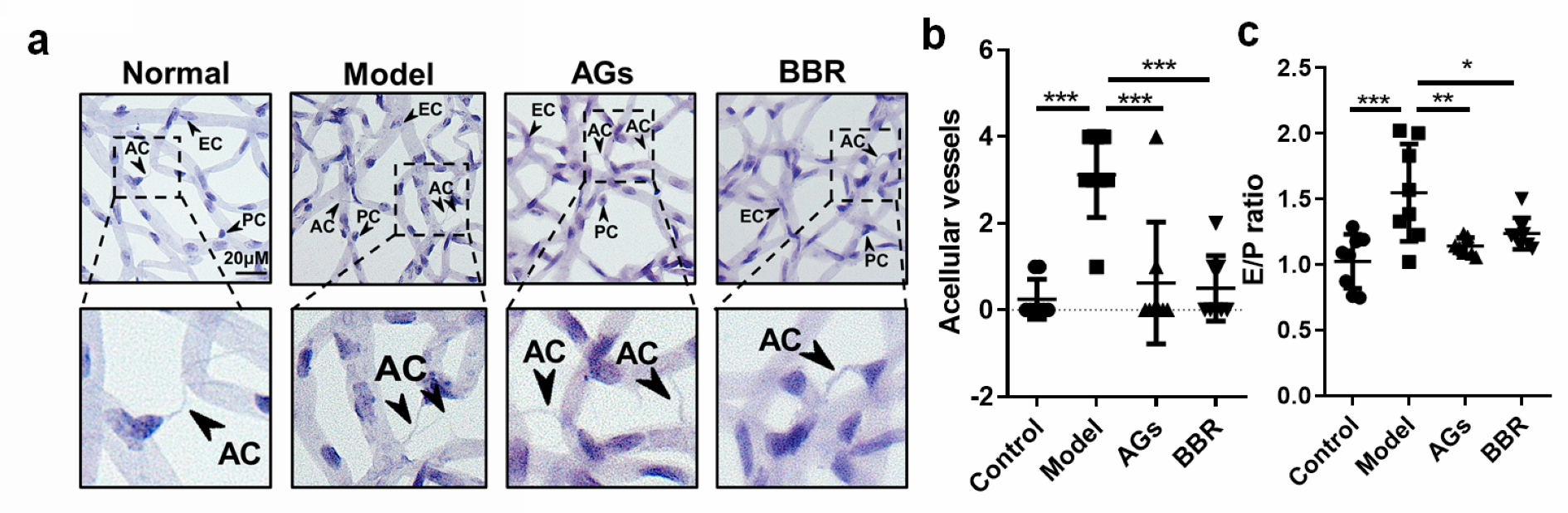


**Figure S2 Berberine improves retina vasculature in hyperglycemic mice**

**a.** Fresh eyeballs of hyperglycemic mice were fixed in PFA and then retina was isolated. The retina was then digested with 3% trypsin and the retina vasculature was then mounted and stained with hematoxylin-eosin staining. Image was then captured under light microscope. Areas of microscope were randomly selected from individual sample to quantify endothelial cells/pericytes (E/P) and acellular capillaries in each sample. AGs or BBR treatment can significantly suppress the formation of acellular vessels (**b**) and reduce the E/P ratio (**c**) in the retina vasculature. *p<0.05, **p<0.01, ***p<0.001 when compared to model group.


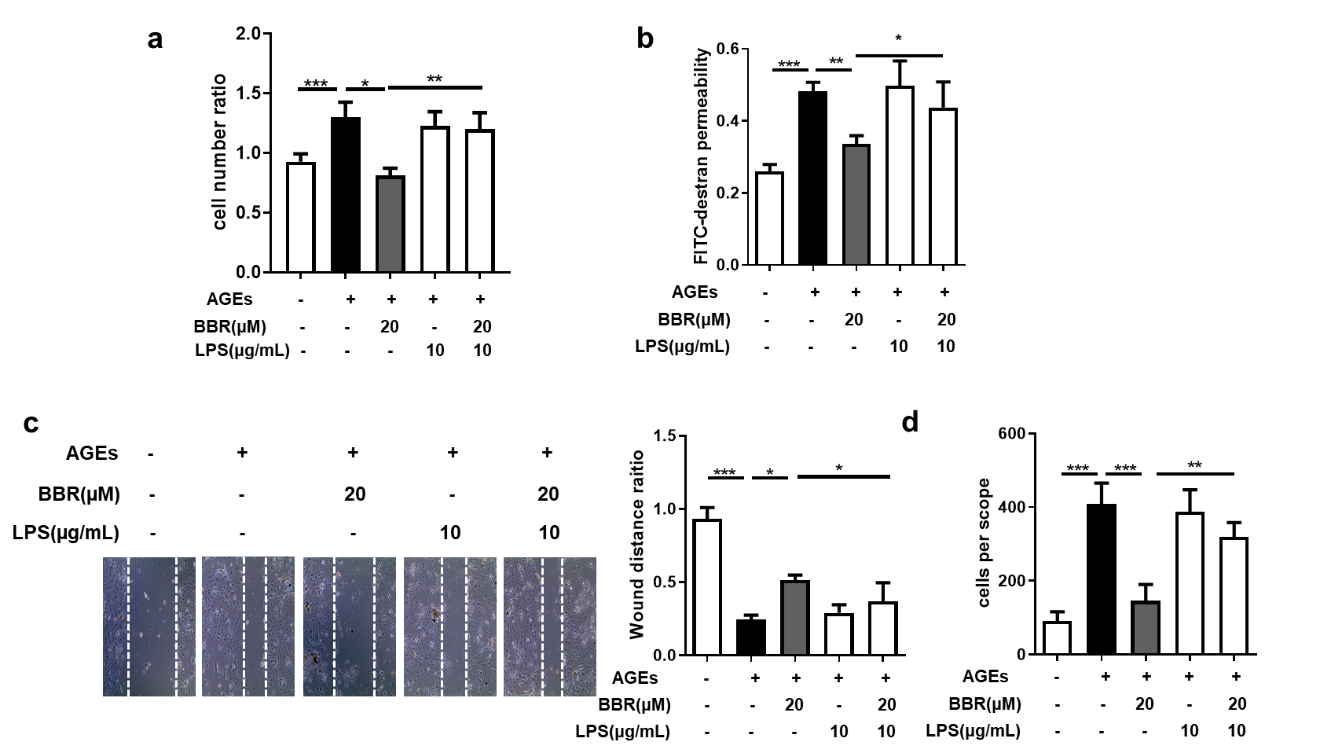


**Figure S3 Activation of TLR4 by LPS restored inhibition of endothelial activation by berberine**

**a.** HRECs was pre-treated with 100µg/ml LPS for 30 min. Medium was then washed away, and cells were treated with BBR in the presence of 20µg/ml AGEs for 72 h. Cell number was counted and the fold increase was calculated. It was shown that LPS pre-treatment significantly restored the cell proliferation of HRECs in the presence of BBR. **b.** HRECs with or without LPS pre-treatment were seeded onto Transwell insert with pore size of 0.4µm (Corning Costar) until full confluence. 200µl ECM with 1% FBS, 25µg/ml FITC-Dextran and 20µg/ml standard AGEs alone or in combination with BBR (4µM or 20µM) were applied to the top inserts respectively, whereas 900 µl ECM with 1% FBS were added in the lower chambers for 24 h incubation. Medium at upper and lower chambers was then collected for measurement of fluorescence with excitation at 494nm and emission at 518nm. The leakage of FITC-Dextran was calculated. It was shown that LPS pre-treatment significantly restored the FITC-Dextran leakage through HRECs monolayer in the presence of BBR; **c.** HRECs with or without LPS pre-treatment were seeded with full confluence and a line was scraped at the middle of culture wells. Cells were then incubated with 20µg/ml AGEs in combination with various treatment for 24 h. The imaged was captured and wound closure rate was calculated. It was shown that LPS pre-treatment significantly restored the wound closure of HRECs monolayer in the presence of BBR; **d.** HRECs with or without LPS pre-treatment were seeded onto the Transwell inserts (pore size as 8µm) supplemented with 20µg/ml AGEs in combination with various treatments. Cells passing through the transwell within 4 h were stained with crystal violet and quantified under light microscope. LPS pre-treatment significantly restored the migration of HRECs monolayer in the presence of BBR. *p<0.05, **p<0.01, ***p<0.001 when compared to AGEs-treated groups in cell experiments.


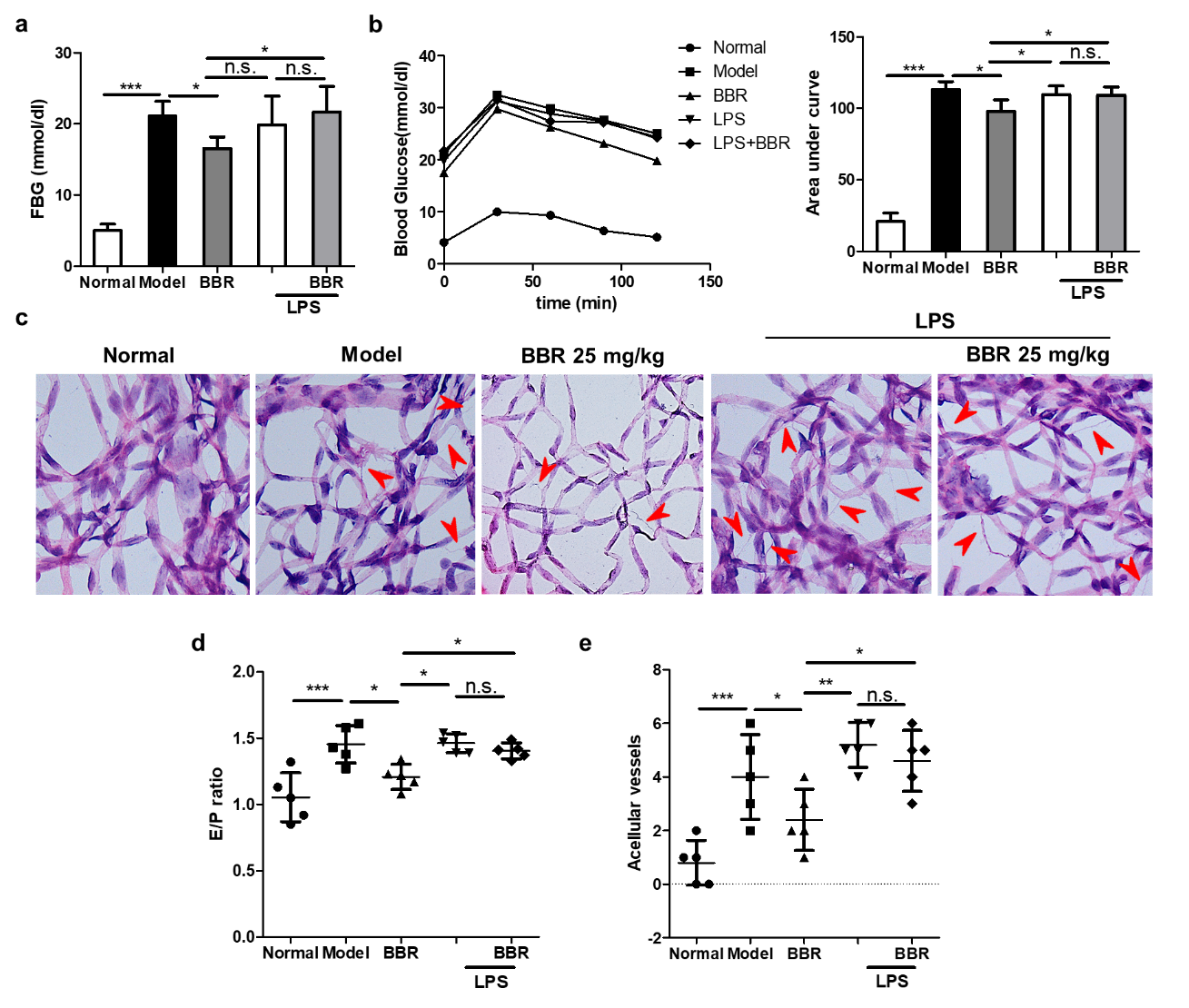

**Figure S4 Supplementation of LPS attenuated the improvement of berberine on hyperglycemia and retina vasculature in hyperglycemic mice**

**a.** Hyperglycemic mice (n=5) was i.p. injected with single dose of LPS (3mg/kg) right before 25mg/kg BBR treatment for 8 weeks. FBG was measured at the end of treatment. It was shown that LPS pre-treatment can significantly abolished the improvement of FBG in BBR-treated hyperglycemic mice; **b.** At the end of 8-week treatment, hyperglycemic mice were starved for 5 h and 2 g/kg glucose was i.p. injected to the mice. the blood glucose was tested at 0, 30, 60, 90 and 120 min post glucose injection. Curve was drawn and the area under curve (AUC) was quantified. It was shown that LPS pre-treatment can significantly abolished the improvement of glucose tolerance in BBR-treated hyperglycemic mice; **c.** Fresh eyeballs of hyperglycemic mice were fixed in PFA and then retina was isolated. The retina was then digested with 3% trypsin and the retina vasculature was then mounted and stained with hematoxylin-eosin staining. Image was then captured under light microscope. Areas of microscope were randomly selected from individual sample to quantify endothelial cells/pericytes (E/P) and acellular capillaries in each sample. It was shown that LPS pre-treatment can significantly abolished the improvement of the formation of acellular vessels (**b**) and reduce the E/P ratio (**c**) in the retina vasculature. *p<0.05, **p<0.01, ***p<0.001 when compared to model group.


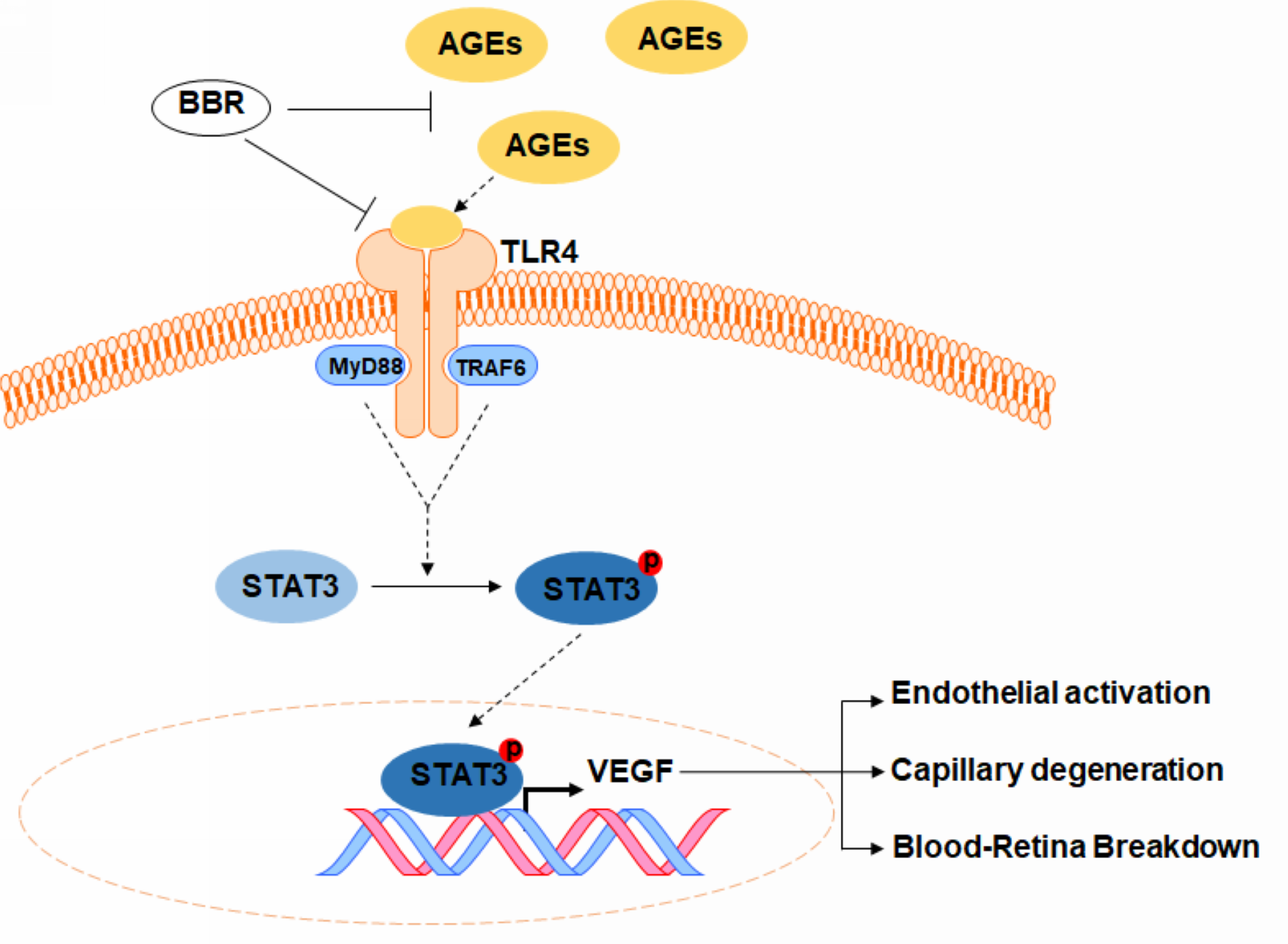


**Figure S5 Schematic presentation of the regulatory mechanisms of BBR in diabetic retinopathy**

**Reference**

1. Diogo CV, Machado NG, Barbosa IA, Serafim TL, Burgeiro A, Oliveira PJ. Berberine as a promising safe anti-cancer agent - is there a role for mitochondria? *Curr Drug Targets.* 2011; **12**(6):850-9.

2. Lan J, Zhao Y, Dong F, Yan Z, Zheng W, Fan J, Sun G. Meta-analysis of the effect and safety of berberine in the treatment of type 2 diabetes mellitus, hyperlipemia and hypertension. *J Ethnopharmacol.* 2015; **161**:69-81.

3. Pang B, Zhao LH, Zhou Q, Zhao TY, Wang H, Gu CJ, Tong XL. Application of berberine on treating type 2 diabetes mellitus. *Int J Endocrinol.* 2015; **2015**:905749.

4. Yin J, Xing H, Ye J. Efficacy of berberine in patients with type 2 diabetes mellitus. *Metabolism.* 2008; **57**(5):712-7.

5. Zhang Q, Xiao X, Li M, Li W, Yu M, Zhang H, Ping F, Wang Z, Zheng J. Berberine moderates glucose metabolism through the GnRH-GLP-1 and MAPK pathways in the intestine. *BMC Complement Altern Med.* 2014; **14**:188.

6. Chueh WH, Lin JY. Protective effect of berberine on serum glucose levels in non-obese diabetic mice. *Int Immunopharmacol.* 2012; **12**(3):534-8.

7. Sun SF, Zhao TT, Zhang HJ, Huang XR, Zhang WK, Zhang L, Yan MH, Dong X, Wang H, Wen YM, Pan XP, Lan HY, Li P. Renoprotective effect of berberine on type 2 diabetic nephropathy in rats. *Clin Exp Pharmacol Physiol.* 2015; **42**(6):662-70.

8. Ni WJ, Ding HH, Tang LQ. Berberine as a promising anti-diabetic nephropathy drug: An analysis of its effects and mechanisms. *Eur J Pharmacol.* 2015; **760**:103-12.

9. Tian P, Ge H, Liu H, Kern TS, Du L, Guan L, Su S, Liu P. Leukocytes from diabetic patients kill retinal endothelial cells: effects of berberine. *Mol Vis.* 2013; **19**:2092-105.

10. Zhang C, Wang N, Tan HY, Guo W, Chen F, Zhong Z, Man K, Tsao SW, Lao L, Feng Y. Direct inhibition of the TLR4/MyD88 pathway by geniposide suppresses HIF-1alpha-independent VEGF expression and angiogenesis in hepatocellular carcinoma. *Br J Pharmacol.* 2020; **177**(14):3240-57.
